# Mechanical Characterization of Intermaxillary Orthodontic Elastics: Energy-Based Metrics and Clinical Guidance

**DOI:** 10.3390/jfb17030117

**Published:** 2026-03-01

**Authors:** Pedro Antunes, Catarina Oliveira, Mariana Santos, Carlos Miguel Marto, Luís Vilhena, Amílcar Ramalho, Inês Francisco, Francisco Vale

**Affiliations:** 1 Centre for Mechanical Engineering, Materials and Processes (CEMMPRE), Advanced Production and Intelligent Systems (ARISE), University of Coimbra, 3004-504 Coimbra, Portugal; vale.antunes@ua.pt (P.A.); cmiguel.marto@uc.pt (C.M.M.); luisvilhena@gmail.com (L.V.); amilcar.ramalho@dem.uc.pt (A.R.); fvale@fmed.uc.pt (F.V.); 2 Centre for Mechanical Technology and Automation (TEMA), Department of Mechanical Engineering, Campus Universitário de Santiago, University of Aveiro, 3810-193 Aveiro, Portugal; 3 Institute of Orthodontics, Faculty of Medicine, University of Coimbra, 3000-075 Coimbra, Portugal; catarinafoliveira6@gmail.com (C.O.); mariana0santos00@gmail.com (M.S.); 4 Coimbra Institute for Clinical and Biomedical Research (iCBR), Area of Environment Genetics and Oncobiology (CIMAGO), Faculty of Medicine, University of Coimbra, 3000-075 Coimbra, Portugal; 5 Laboratory for Evidence-Based Sciences and Precision Dentistry, University of Coimbra, 3000-075 Coimbra, Portugal; 6 Clinical Academic Center of Coimbra (CACC), Coimbra University Hospital, 3004-561 Coimbra, Portugal; 7 Centre for Innovative Biomedicine and Biotechnology (CIBB), University of Coimbra, 3004-504 Coimbra, Portugal; 8 Institute of Integrated Clinical Practice, Faculty of Medicine, University of Coimbra, 3004-531 Coimbra, Portugal; 9 Institute of Experimental Pathology, Faculty of Medicine, University of Coimbra, 3004-531 Coimbra, Portugal

**Keywords:** orthodontics, intermaxillary elastics, mechanical characterization

## Abstract

**Background**: Intermaxillary elastics are widely used in orthodontics to deliver controlled forces for malocclusion correction, aiding in the correction of anteroposterior, vertical, or transverse problems. Despite their clinical relevance, comprehensive mechanical characterization remains limited. **Objective**: This study aimed to evaluate the mechanical properties of nine types of intermaxillary elastics available on the market to guide evidence-based clinical selection. **Methods**: Elastics were tested under uniaxial tensile loading following ISO 37:2011 and ISO 21606:2007, with six replicates per type. Load–displacement and stress–strain responses were analyzed, measuring peak force, elongation at rupture, work-to-rupture, and specific rupture work. Non-linear behavior was modeled using cubic polynomial regression, and normalized stress–strain curves enabled intrinsic material comparisons. One-way ANOVA with post-hoc tests assessed differences among elastics. **Results**: All elastics displayed characteristic non-linear elastomeric responses. Functional grouping distinguished short-displacement/high-stiffness, intermediate-displacement/moderate-stiffness, and long-displacement/high-capacity bands. Work-to-rupture, specific rupture work, and normalized stress–strain metrics varied significantly, reflecting differences in energy absorption and force delivery (*p* < 0.05). **Conclusions**: Mechanical characterization, including energy-based descriptors and normalized stress–strain analysis, supports informed elastic selection, enhancing orthodontic treatment predictability and patient safety.

## 1. Introduction

Orthodontic elastics have long been a cornerstone of clinical practice, providing a simple yet highly effective means of achieving precise tooth movements and correcting interarch discrepancies. Introduced in the late 19th century and only widely adopted in the 1960s, these removable elastics have since evolved significantly in both material composition and application techniques [1,2]. Their inherent properties—such as high flexibility and the capacity to deliver continuous force over time—along with their affordability and patient-friendly application, render them a highly effective tool in achieving successful orthodontic outcomes [3,4,5].

They can be classified based on their site of action as either extraoral or intraoral. Extraoral elastics function in conjunction with external orthodontic appliances and may apply either dental or skeletal effects. Intermaxillary elastics operate between opposing dental arches, distributing force between the maxilla and the mandible [6]. Intermaxillary elastics constitute a fundamental biomechanical resource for achieving controlled orthodontic tooth movement, particularly in procedures involving extrusion, vertical alveolar development, and space closure. Their calibrated force application enables gradual extrusion with reduced periodontal risk, while simultaneously promoting alveolar bone remodeling—an essential factor in pre-implant site preparation and optimization of osseointegration. Moreover, intermaxillary elastics contribute significantly to molar uprighting and occlusal plane modulation. Through controlled and strategic force distribution, they facilitate axial correction of tipped molars, enhance anchorage control, and improve occlusal relationships [7,8,9].

There are two types of orthodontic elastics: latex or synthetic. Latex elastics, obtained from plant extracts, were the original and remain the most commonly used type, whereas synthetic elastics are produced chemically from coal, petroleum and some vegetable alcohols and are preferred in patients with latex allergies [5,10,11]. The mechanical characteristics of orthodontic elastics directly influence their clinical performance, particularly parameters such as thickness (which determines their force classification, e.g., 2 ozf or 6 ozf), diameter, cut width, and the inherent properties of the material [5]. Manufacturers generally recommend stretching each elastic to approximately three times its diameter to achieve optimal force levels, requiring clinicians to select the appropriate size and thickness for effective treatment outcomes [12]. In addition, all elastomeric materials experience creep and tension relaxation [5,6,11,12,13], leading to elastic force degradation over time and a reduced ability to sustain the intended tooth movement. This phenomenon, known as elastic force degradation [4,5], reflects the progressive loss of the material’s ability to compensate for imposed deformation. The degradation process is further intensified by biological, chemical, and physical factors present in the oral cavity, such as saliva composition, pH, nutritional habits and thermocycling, which accelerate the degradation process, as well as by storage conditions, including exposure to light and ambient temperature [1,3,10,11,14]. This degradation occurs rapidly during the first hour of intraoral aging, characterized by a marked reduction in stiffness, followed by a slower decay phase and stabilization after approximately 4 h. This progression reflects the typical viscoelastic behavior of intermaxillary elastics when strained and exposed to the oral environment [15].

Several studies investigated the mechanical behaviour of orthodontic elastics, as understanding force degradation is closely related to the success of orthodontic treatment. Most research has focused on latex elastics under standardized, static conditions, while limited studies have assessed synthetic materials or actual elongation distances, and more advanced mechanical descriptors such as energy absorption and stress-strain behavior remain underreported [16,17]. Saccomanno et al. conducted a systematic review encompassing several in vitro studies on intraoral orthodontic elastics, highlighting the considerable variability in force degradation across different brands and types, and emphasizing the need for standardized mechanical characterization protocols that extend beyond force decay to include stiffness, energy dissipation, and elastic response under functional loading [3]. Energy-based metrics, including work to rupture (W_R_) and specific rupture work, offer critical insight into the capacity of intermaxillary elastics to sustain forces under prolonged or high-strain conditions, which is essential for clinical applications such as tooth extrusion, space closure, and occlusal plane modulation [7]. In orthodontic biomechanics, force delivery, decay, and load stability to dental and periodontal structures are critical. Kanchana P et al. showed that natural latex elastics from four manufacturers exhibited up to 30% initial force loss in wet conditions, followed by minimal reduction over three days. Despite minor deviations from the standard force–extension index, their performance was clinically acceptable, though inter-manufacturer variability highlights the importance of careful material selection [10].

A deeper understanding of the mechanical properties of elastics—including the force generated and the way it degrades over time—enables clinicians to select the most suitable type for each patient. In contrast to previous studies that focused primarily on initial force and force degradation, the present work adopts a comprehensive approach, evaluating parameters such as maximum elongation at rupture, maximum force sustained, load–displacement relationship, work to rupture, stress, and specific rupture work. Unlike previous studies focusing primarily on initial force and force degradation, this study integrates energy-based and stress-strain descriptors to connect material properties with orthodontic biomechanics. By employing polynomial regression models and analyzing statistical variations, this study provides a robust quantitative framework for comparing elastics of different cross-sectional dimensions and material compositions, supporting clinical decision-making and the development of a device to assist orthodontists in selecting intermaxillary elastics.

The present study aims to deepen the understanding of the mechanical properties of intermaxillary elastics by evaluating critical parameters that directly influence their clinical effectiveness [18]. Unlike previous studies, which primarily focused on initial force and force degradation over time [16], this research incorporates a comprehensive analysis of mechanical parameters, specifically:

- Maximum elongation at rupture;
- Maximum force sustained;
- Load–displacement relationship (N/mm);
- Work to rupture (J/m^2^);
- Stress (N/mm^2^);
- Specific rupture work (J·m^−2^).

## 2. Materials and Methods

The experimental methodology was conducted in accordance with ISO 37:2011 [19], which outlines procedures for determining tensile properties of elastomeric materials. Furthermore, guidelines from ISO 21606:2007 [20], addressing physical properties of orthodontic materials, were followed to ensure clinical relevance of the obtained data. Previous studies, such as those by Dittmer et al. [21], have similarly employed ISO 21606:2007 [20] to assess tensile properties of orthodontic elastomeric chains, underscoring the importance of normative standards for achieving comparable and clinically applicable results.

A total of nine distinct types of latex intermaxillary elastics (E-1 to E-9), each corresponding to a different commercial product and defined by specific commercial designations and manufacturer-reported mechanical properties [22], were included in this study, with six independent specimens tested for each elastic type (*n* = 6). All elastics were tested within the manufacturer-specified expiry period, and all specimens belonged to the same production lot to ensure consistency and to minimize variability associated with inter-lot differences and material aging. These elastics were selected to ensure a wide range of force levels, sizes, and material properties, providing a comprehensive understanding of their mechanical behavior [23]. The detailed specifications of the tested elastics are summarized in Table 1.

It is important to note that the values presented in Table 1 were obtained from the manufacturers’ specifications. However, the information provided did not clarify what each measurement represents in terms of performance or testing conditions. Notably, the use of “ozf” (ounce-force) and “gf” (gram-force) as measurements for force is incorrect, as these are units of mass, not force [24]. The values were converted into consistent units (N, gf, mm) to allow a direct comparison between the different elastics. These values serve as a reference to validate the relative performance of each elastic during the mechanical tests, without being directly considered as absolute values of performance.

It should be highlighted that only one brand of elastics among those tested provided a quantitative classification of their products, categorizing them as medium, heavy, and strong pull. These classifications were used to guide the selection of elastics for analysis. Additionally, the elastics were categorized by their sizes, specified in both inches (1/8”, 3/16”, and 1/4”) and corresponding diameters (3.2 mm, 4.8 mm, and 6.4 mm), as indicated on the packaging [25].

The elastics were obtained in their original packaging, properly sealed, and within their expiration date, ensuring the integrity of the material [26]. Each elastic was classified based on its force level and size, as indicated by the manufacturer’s specifications [27]. These specifications were used as a reference during the experimental procedures to ensure the consistency of the results [1]. Additionally, all elastics were visually inspected before testing to confirm their integrity and absence of any defects, such as visible surface damage or deformation [28]. This preliminary inspection was essential to maintain the validity of the mechanical tests conducted in the subsequent sections [29].

All tested elastics were measured using an optical microscope (HR-5000, 3D digital microscope, Hirox USA Inc., Hackensack, NJ, USA), which allowed precise determination of their internal diameter (ϕ int), external diameter (ϕ ext), height (h), and thickness (t) [30]. The cross-sectional area reported as Section (A_0_) in Table 2 was calculated as A_0_ = t × h, in [mm^2^]. The measured values presented in Table 2 provide an accurate representation of the physical dimensions of the elastics used in this study, ensuring consistency in the analysis of their mechanical properties [31]. These measurements are crucial for the mechanical characterization of the elastics because they directly influence their stress distribution, load-bearing capacity, and deformation behavior during application [32].

**Figure 1:**
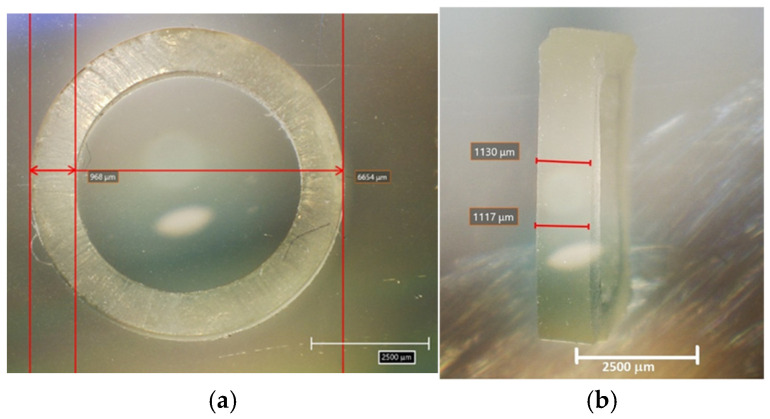
Example of measurement procedure for each elastic in the study: (**a**) Precise measurements of each elastic’s internal and external diameters; (**b**) Measurements of the elastic’s thickness and height.

As an example of the procedure applied to each elastic, Figure 1a,b present the images obtained using the optical microscope for one of the tested elastics, illustrating the precise measurements of each elastic’s internal and external diameters, thickness, and height. This approach was consistently applied to all tested elastics, ensuring the reliability and repeatability of the measurements.

Initially, each elastic was fixed onto a custom-designed hook, and tensile stretching was applied. Throughout the stretching process, both the released force and the elongation were continuously measured. All specimens were conditioned for 24 h prior to testing under controlled laboratory conditions in accordance with ISO 291:2008 [31]. Tensile tests were performed at 22 ± 2 °C and 54 ± 5% relative humidity. These environmental conditions were maintained constant throughout all experimental procedures. Testing procedures were carried out in the laboratory of the Department of Mechanical Engineering at the University of Coimbra.

Each elastic ring was carefully mounted on a universal testing machine in accordance with ISO 37:2011 [19]. Tensile testing was conducted with a clamp displacement rate of 3.3 mm/min. The data acquisition system operated at a sampling rate of 100 Hz, continuously capturing force and displacement values throughout each test. All calculations were standardized across specimens, and average values were computed for each elastic type to facilitate comparison.

It is important to emphasize that the mechanical behavior of intermaxillary elastics is non-linear, especially beyond the initial loading phase. Therefore, the concept of a constant elastic (Young’s) modulus is not strictly applicable throughout the entire deformation range [32,33,34,35,36,37,38,39,40]. In this context, any reference to the elastic modulus should be interpreted as an apparent modulus calculated over a limited, near-linear portion of the load–displacement curve. This approach allows for comparative assessments but does not imply ideal elastic behavior.

### 2.1. Tensile Testing

The tensile properties of the elastics were evaluated using an Instron universal testing machine, following the guidelines of ISO 37:2011 [19]. Tests were conducted at a crosshead speed of 3.3 mm/min. The acquired data included crosshead displacement (mm), force sustained (N), and time (s) [30]. The tests were performed at a constant crosshead speed of 3.3 mm/min, following the constant-rate-of-extension principle defined in ISO 37:2011 [19]. This rate was selected to ensure controlled quasi-static loading conditions suitable for comparative mechanical characterization of elastomeric materials.

Tensile tests were conducted using a custom cylindrical pin (hook-type) mounting configuration. The pins were fabricated from AISI 316 stainless steel, with a wire diameter of 1.58 mm, and featured a polished, smooth surface finish to minimize frictional effects and stress concentrations at the contact interface. The pins were carefully aligned coaxially to ensure uniaxial tensile loading conditions, thereby avoiding unintended torsional or bending contributions that could influence the measured mechanical response.

Each elastic ring was mounted around the two cylindrical pins, ensuring that no twist or torsion was present before loading. The crosshead displacement was then gradually increased until a standardized preload of 0.05 N was detected by the load cell. This preload value was selected to exceed the instrument resolution threshold while remaining well below the initial linear deformation region of the elastic, thereby eliminating slack without inducing measurable pre-strain.

The application of this controlled preload also ensured stable seating of the elastic on the pins and prevented slip during testing. No slippage was observed, as confirmed by the repeatability of the initial loading slope across replicates and by post-test visual inspection.

Once the 0.05 N preload was achieved, the displacement was slightly reversed to eliminate seating effects, and the separation between pins was measured using a digital caliper. This measured separation was recorded as the initial gauge length (L_0_) for that specimen. Because ring elastics do not have a predefined gauge length, this procedure provides a repeatable mechanical definition of L_0_ specific to each elastic.

After recording L_0_, both the load cell and displacement readings were reset to zero before initiating each tensile test.

**Figure 2:**
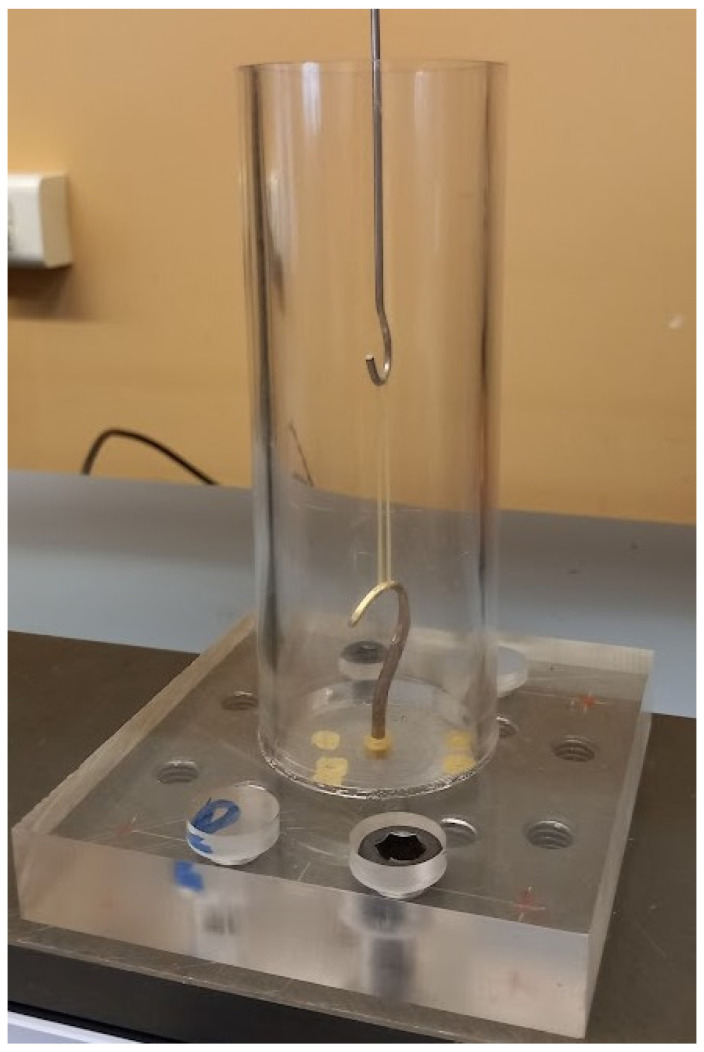
Photographic view of the hook-type cylindrical pin gripping configuration used in the tensile tests, ensuring coaxial alignment and uniaxial loading conditions.

A photographic image of the experimental gripping configuration has been added to enhance clarity and ensure full reproducibility of the setup. The image presents the actual hook-type cylindrical pin mounting system used during the tensile tests, allowing direct visualization of the alignment conditions, geometric arrangement, and boundary constraints applied to the specimens. This addition improves the transparency of the experimental methodology and facilitates accurate replication of the testing configuration, Figure 2.

Because intermaxillary elastics are closed-ring specimens without a predefined gauge length, strain was defined based on the hook-to-hook (pin-to-pin) separation during tensile loading.

The initial gauge length (L_0_) was defined as the separation between cylindrical pins measured under a standardized preload of 0.05 N. Engineering strain (ε) was calculated as Equation (1):

(1) $$\varepsilon =\frac{L-L_{0}}{L_{0}}$$

where L is the instantaneous pin separation and L_0_ is the initial gauge length.

All stress–strain curves presented in this study are based on engineering strain.

Because the ring elastic forms two load-bearing segments in parallel between the pins, the applied tensile force is supported by two identical cross-sectional areas. Therefore, engineering stress (σ) was calculated as Equation (2):

(2) $$\sigma =\frac{F}{{2A}_{0}}\;$$

where A_0_ is the average initial cross-sectional area of a single segment of the elastic.

The cross-sectional areas used in the calculations correspond to the values reported in Table 2 (Section [mm^2^]), which were determined from optical microscopy measurements of elastic thickness and height prior to tensile testing. This procedure ensures a consistent and reproducible definition of both strain and stress for ring elastics under uniaxial tensile loading.

No mechanical preconditioning cycles were applied prior to tensile testing. Each elastic specimen was mounted and subjected directly to monotonic uniaxial loading until rupture.

The aim of the present study was to characterize the ultimate tensile response and energy absorption capacity of intermaxillary elastics under controlled laboratory conditions. Therefore, elastics were tested only once, without prior cyclic loading.

### 2.2. Energy Normalization

Rupture was defined as complete specimen separation, identified by an abrupt force drop to near-zero values and confirmed by visual inspection.

No mechanical preconditioning cycles were applied prior to tensile testing. Each elastic specimen was mounted and subjected directly to monotonic uniaxial loading until rupture.

The work to rupture (W_R_) was calculated as the area under the force–displacement curve up to rupture, as shown in Equation (3):

(3) $$W_{R}=\int_{0}^{\delta_{R}}F\left(\delta \right)d\delta$$

To enable geometry-normalized comparison, rupture work was divided by the initial cross-sectional area, as shown in Equation (4):

(4) $$\frac{W_{R}}{A_{0}}$$

This parameter, referred to as specific rupture work, is expressed in J·m^−2^ and represents the energy absorbed per unit cross-sectional area up to rupture.

Six independent specimens from the same production lot were tested for each elastic type (E-1 to E-9). All reported quantities follow SI units unless explicitly stated on manufacturer labels.

**Figure 3:**
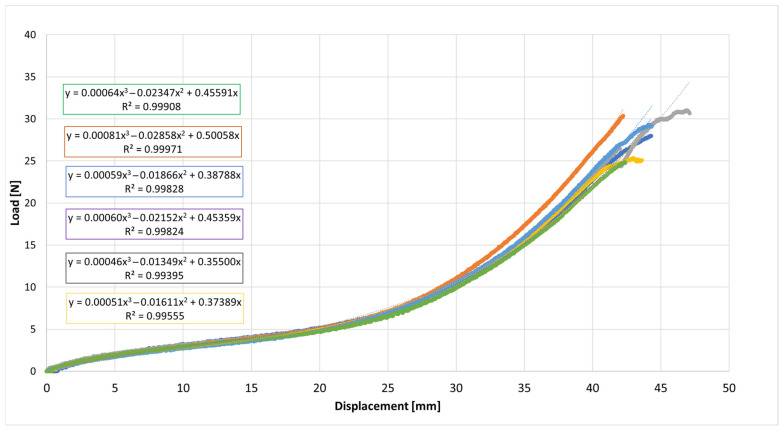
Representative load–displacement curve of elastic E-1 illustrating the typical non-linear elastomeric response and the area under the curve used for work-to-rupture (WR) calculation.

The acquired data were exported, and six independent specimens from the same lot were tested for each elastic type (E-1 to E-9). Two types of graphs were plotted: (i) Load versus Displacement (N vs. mm) (Figure 3). This graph allows the determination of W_R_, calculated as the area under the curve. W_R_ represents the total energy absorbed by the elastic until rupture, providing insights into the durability and resilience of the material; (ii) Stress versus Strain: This graph normalizes the mechanical response by the cross-sectional area, allowing for direct comparison between elastics. The area under this curve is calculated as Tenacity, representing the energy absorbed per unit area [12].

The load–displacement curves exhibited the characteristic nonlinear elastomeric response: an initial quasi-linear region followed by progressive stiffening as elongation increases, consistent with strain-induced hardening of cross-linked rubber networks [31,33].

To describe the experimental curves, a third-order polynomial was fitted to each dataset, as shown in Equation (5):

F(x) = a∙x^3^ + b∙x^2^ + c∙x (5)

where F is the load (N) and x is the displacement (mm).

The cubic polynomial fits achieved high coefficients of determination (R^2^ ≈ 0.995–0.999), indicating excellent agreement with the experimentally measured stress–strain data within the tested deformation range.

The fitted functions were obtained from the mean stress–strain curves calculated for each elastic type within the same production lot. To ensure strict adherence to the experimentally validated domain, the fitted curves were truncated at the smallest maximum deformation observed among the tested specimens. No extrapolation beyond measured data was performed.

The regression functions serve solely as descriptive mathematical representations of the recorded nonlinear response and are not intended as constitutive material models. Accordingly, no mechanistic interpretation is attributed to individual polynomial coefficients.

Across the six elastic bands, the individual load–displacement curves largely overlap up to about 25 mm of displacement, indicating highly consistent mechanical performance. Beyond ~35 mm, small divergences appear in the high-strain region, where some curves exhibit slightly higher stiffness. The maximum difference in load at large displacements is on the order of 5–10%, which is acceptable for elastomeric products manufactured in separate batches. This limited variability suggests that production tolerances and material homogeneity were well controlled.

Overall, the results demonstrate that the six tested elastic bands share a comparable mechanical response, with only minor variations in the nonlinear stiffening region. Such consistency supports the use of a representative mean curve for each elastic type, accompanied by dispersion bands (e.g., ±1 standard deviation) to quantify variability. This average representation is valuable for subsequent analyses of mechanical properties such as initial stiffness, energy absorbed up to failure, and strain energy density to rupture.

W_R_, representing the total energy absorbed by an elastic until rupture, is analogous to the parameter known as specific rupture work in materials science. Toughness is defined as the area under the stress-strain curve up to the point of failure. In the context of orthodontic materials, Ahrari et al. evaluated the specific rupture work of elastomeric ligatures, noting significant decreases after 28 days of immersion in a simulated oral environment [1]. This suggests that specific rupture work, or W_R_, is a critical parameter in assessing the durability and energy absorption capacity of orthodontic elastics.

To compare the mechanical behavior of the six specimens tested for each type of elastic (E-1 to E-9) under tensile loading and to support the definition of their most suitable clinical application, six independent force–displacement tests were performed per elastic type (*n* = 6). The raw curves obtained from individual tests were interpolated onto a common displacement grid to enable point-wise comparison across specimens.

**Figure 4:**
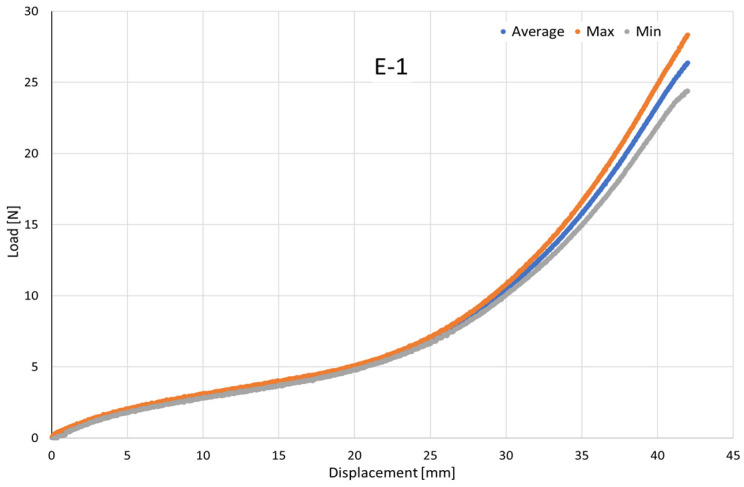
Representative load–displacement response of elastic band E-1 showing the mean curve (blue), together with the maximum (orange) and minimum (grey) envelopes obtained from six replicate tensile tests.

For each displacement increment, the mean force was computed as the arithmetic average of the six tests, while the experimental scatter was quantified through the sample standard deviation. The resulting mean curve represents the characteristic tensile response of each elastic band, whereas the ±1 σ envelope illustrates the dispersion among specimens (Figure 4). To provide an analytical description of each mean response, a cubic regression was fitted to the averaged data, yielding a compact equation suitable for comparative assessment and further modelling of the elastic bands’ mechanical performance in service.

The maximum and minimum envelopes obtained from the six replicates were plotted with the mean curve to show the force variability at each displacement, allowing clinicians to estimate both the expected force and its possible range, thereby supporting more predictable and safer selection of intermaxillary elastics.

**Figure 5:**
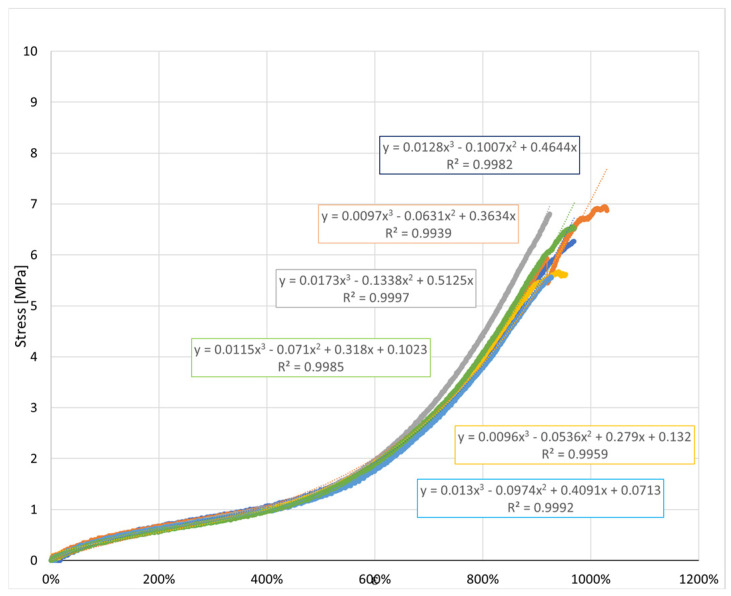
Stress–strain curves of the tested elastics with cubic regression fits and ISO 37:2011 [20] tensile parameters.

Figure 5 presents the stress–strain curves of the six types of orthodontic elastics tested in uniaxial tension according to the procedures described in ISO 37:2011 [19]. All samples exhibited the typical nonlinear elastomeric behavior, with tensile stress increasing progressively with elongation. In addition to the overall curve profile, relevant standardized parameters, such as tensile stress at specified elongations, elongation at break, and tensile set after rupture, were determined as defined in the standard.

Beyond the standard tensile parameters defined by ISO 37:2011 [19], the strain energy density to rupture—calculated as the integral of the stress–strain curve up to rupture—was evaluated to quantify the energy absorbed per unit cross-sectional area. This metric provides complementary insight into the energy-absorption capacity of the elastics and is particularly relevant for clinical applications where sustained energy delivery is required. Although the term strain energy density to rupture is seldom reported in orthodontic literature, its physical meaning aligns with the concepts of material-specific rupture work. Incorporating this parameter, alongside the ISO-defined properties, offers a more comprehensive mechanical characterization of the elastics.

All tensile tests were performed under controlled laboratory conditions (22 °C, 54% relative humidity) in accordance with ISO 37:2011 [19] to ensure reproducibility and comparability of results. Experimental data were statistically processed to compute the mean values and standard deviations for each reported property.

### 2.3. Statistical Analysis of Results

To determine whether the apparent differences among elastic bands were statistically meaningful rather than artifacts of experimental scatter, a one-way analysis of variance (ANOVA) was conducted for each mechanical outcome reported in Table 3, using elastic designation (E-1 to E-9) as the grouping factor and *n* = 6 independent specimens per group. The outcomes analyzed were: specific rupture work (J·m^−2^), displacement at peak load (mm), peak load (N), strain at peak (%), and peak stress (MPa). These endpoints complement the standardized tensile descriptors recommended for elastomeric materials and orthodontic auxiliaries [19,20].

#### 2.3.1. Assumption Checks

Before each ANOVA, normality of residuals was assessed using the Shapiro–Wilk test and visual inspection of Q–Q plots, and homogeneity of variances was evaluated using Levene’s test. When homogeneity assumptions were satisfied, classical one-way ANOVA was applied. If variance homogeneity was violated, Welch’s ANOVA was used. In cases of pronounced non-normality not corrected by simple transformations (e.g., logarithmic transformation for strictly positive measures), the Kruskal–Wallis test was used as a non-parametric alternative. This workflow follows established statistical practice for mechanical testing of polymeric and orthodontic materials [34].

For outcomes with significant omnibus tests (*p* < 0.05), Tukey’s HSD post-hoc comparisons (for classical ANOVA) or Games–Howell tests (for Welch’s ANOVA) were used to identify significant pairwise differences while controlling the family-wise error rate. Alongside the *p*-values, effect sizes (ω^2^ for omnibus ANOVA and Hedges’ g for pairwise comparisons) and 95% confidence intervals for mean differences were reported to quantify the magnitude of differences and their potential clinical relevance.

To complement inferential analysis, box-and-whisker plots with jittered points were generated for each outcome to visualize dispersion, variability, and potential outliers, consistent with reporting recommendations in orthodontic materials research [1,3,4,36].

Dimensional measures (L_0_, diameter, and section) were treated as constant within each product due to measurement constraints. This does not affect ANOVA on geometry-independent outcomes (e.g., specific rupture work in J·m^−2^, peak stress in MPa) but should be acknowledged as a limitation for analyses involving geometry-dependent quantities. The normalization to stress–strain representation mitigates size effects and supports fair between-product comparisons.

Full post-hoc pairwise comparison tables (adjusted *p*-values and 95% confidence intervals) are provided in the Supplementary Materials.

#### 2.3.2. Outlier Handling

No data points were excluded from the statistical analysis. All specimens tested (*n* = 6 per elastic type) were included in the analyses. Potential outliers were visually inspected using box-and-whisker plots and standardized residual diagnostics; however, no observations were removed, as all values were consistent with the experimental variability expected for elastomeric materials under tensile loading. This approach avoids post-hoc data manipulation and ensures full transparency and reproducibility of the reported results.

**Figure 6:**
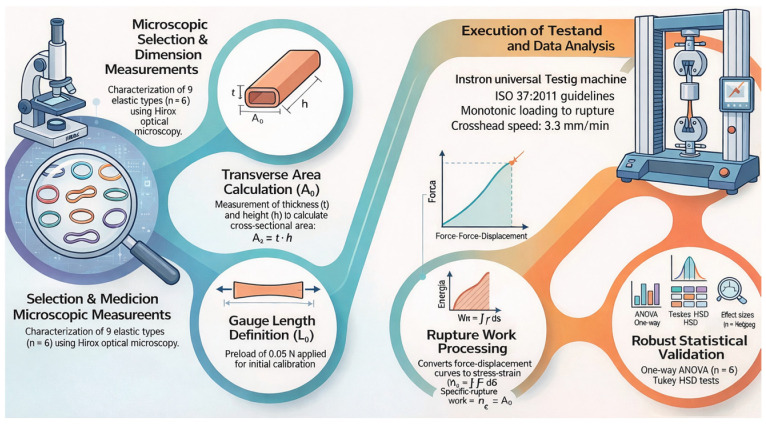
Experimental workflow illustrating the main stages of the study, including microscopic selection of specimens, dimensional measurements (internal and external diameters, thickness, and height), mounting procedure, tensile testing until rupture, calculation of mechanical parameters, and statistical analysis of the results.

To improve methodological transparency and facilitate reproducibility, a schematic flowchart summarizing the experimental workflow is presented in Figure 6. The diagram outlines the sequential steps of the study, including: (i) dimensional characterization by optical microscopy, (ii) specimen mounting and L_0_ definition under standardized preload, (iii) monotonic tensile testing to rupture under controlled quasi-static conditions, (iv) data acquisition and normalization to engineering stress–strain, and (v) statistical analysis and regression modeling.

This structured representation allows readers to clearly follow the progression from specimen preparation to data analysis, enhancing clarity and reproducibility of the experimental protocol.

## 3. Results

This section presents the experimental characterization of the nine intermaxillary elastics (E-1 to E-9) under uniaxial tensile loading, beginning with the raw load–displacement curves from the six independent specimens tested for each elastic type and the corresponding mean curves with their variability envelopes. To allow size-independent comparison, the behavior was also expressed in stress–strain form, and the principal mechanical properties—peak load, peak displacement and stress, work to rupture, and specific rupture work—are summarized in Table 3 (mean ± standard deviation).

Despite the overall similarity in the general shape of the load–displacement and stress–strain curves, clear quantitative differences were observed among the elastics, particularly in the slopes at intermediate elongations and in the maximum tensile stress attained. These variations reflect differences in apparent stiffness and ultimate tensile strength between products. To quantitatively describe the nonlinear mechanical response, cubic polynomial regressions were fitted to the experimental curves. All regressions exhibited high coefficients of determination (R^2^ > 0.99), confirming the suitability of the cubic model to accurately capture the stress–strain behavior of the intermaxillary elastics over the full deformation range.

### 3.1. Statistical Analysis

One-way ANOVA revealed significant differences among elastics for all evaluated endpoints: specific rupture work (J·m^−2^), F(8, 45) = 69.43, *p* = 9.42 × 10^−23^, ω^2^ = 0.910; displacement at peak load (mm), F(8, 45) = 466.97, *p* = 1.19 × 10^−40^, ω^2^ = 0.986; peak load (N), F(8, 45) = 22.19, *p* = 3.09 × 10^−13^, ω^2^ = 0.758; strain at peak (−), F(8, 45) = 353.74, *p* = 5.59 × 10^−38^, ω^2^ = 0.981; and peak stress (MPa), F(8, 45) = 50.60, *p* = 5.78 × 10^−20^, ω^2^ = 0.880. Tukey HSD post-hoc tests (family-wise adjusted *p*-values) identified multiple significant pairwise differences between elastics; full pairwise outputs (adjusted *p*-values and 95% CIs) are provided in the Supplementary Materials.

Post-hoc Tukey HSD comparisons further clarified the magnitude of differences between selected elastics. For peak stress, E-7 showed significantly higher values than E-9 (mean difference = 8.890 MPa, 95% CI [6.822, 10.958], *p* = 5.66 × 10^−15^, g = 20.55). For peak load, E-5 generated significantly higher forces than E-2 (mean difference = 19.902 N, 95% CI [14.221, 25.582], *p* = 2.59 × 10^−13^, g = 6.51). Regarding specific rupture work, E-4 exceeded E-1 (mean difference = 809.174 J·m^−2^, 95% CI [658.954, 959.394], *p* = 5.33 × 10^−15^, g = 10.11). E-6 demonstrated greater extensibility than E-2 in terms of displacement at peak load (mean difference = 51.879 mm, 95% CI [47.874, 55.884], *p* = 5.33 × 10^−15^, g = 14.48). Similarly, E-9 exhibited significantly higher strain at peak compared with E-2 (mean difference = 8.356, 95% CI [7.755, 8.956], *p* = 5.33 × 10^−15^, g = 26.07). Complete post-hoc comparison tables (adjusted *p*-values and 95% confidence intervals) are provided in the Supplementary Materials.

Table 3 summarises the average mechanical properties (mean ± standard deviation) determined for each of the nine intermaxillary elastic bands (E-1 to E-9). The reported parameters include the energy absorbed up to rupture (W_R_), displacement and stress at peak load, peak load, and strain at peak load [4]. These values provide a quantitative overview of the mechanical performance of each elastic type, supporting the subsequent interpretation of the load–displacement and stress–strain curves. This structure allows a comprehensive assessment of the elastics’ mechanical performance, highlighting both the typical response of each band and the experimental scatter within each group. Such information is crucial for clinical application, as it supports the selection of elastics capable of delivering the desired orthodontic force range for specific intermaxillary displacements [36].

### 3.2. Force–Displacement Behaviour of All Elastics

**Figure 7:**
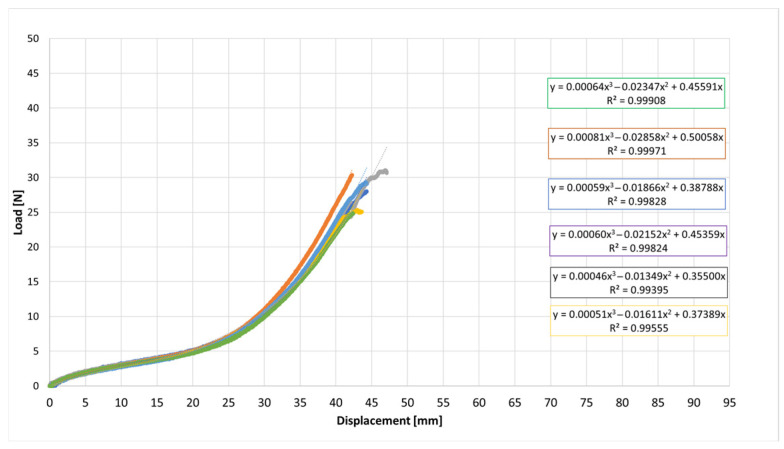
Load–displacement curve for test designation elastic, E-1.

**Figure 8:**
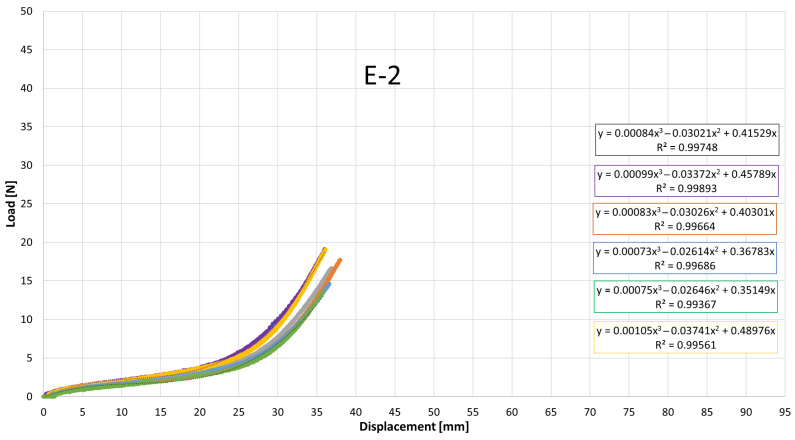
Load–displacement curve for test designation elastic, E-2.

**Figure 9:**
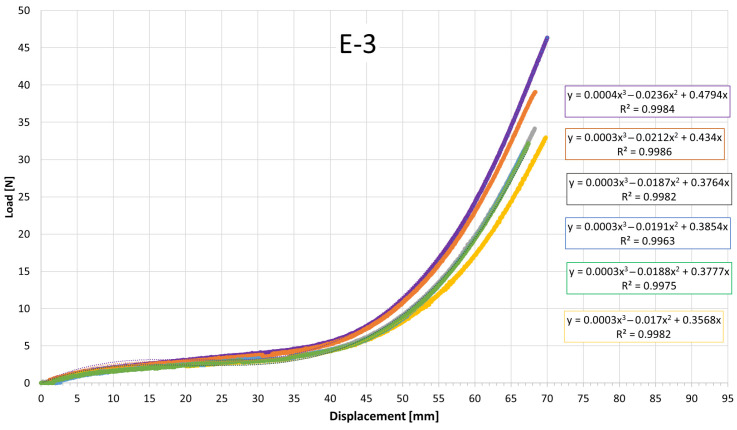
Load–displacement curve for test designation elastic, E-3.

**Figure 10:**
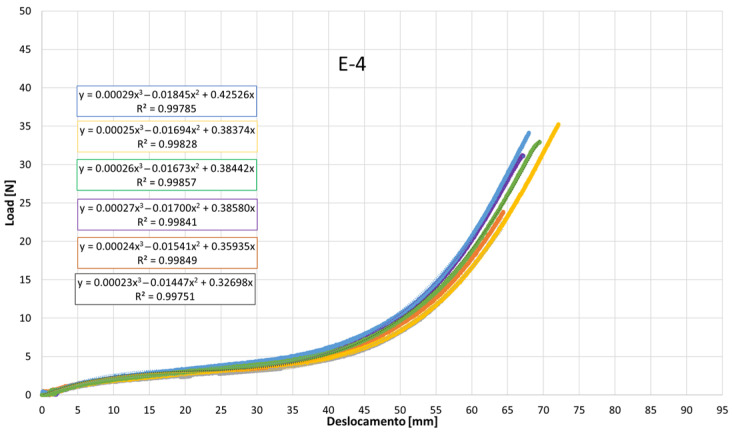
Load–displacement curve for test designation elastic, E-4.

**Figure 11:**
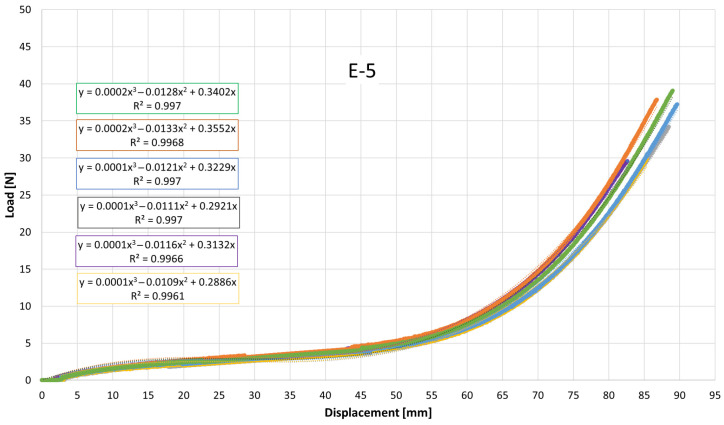
Load–displacement curve for test designation elastic, E-5.

**Figure 12:**
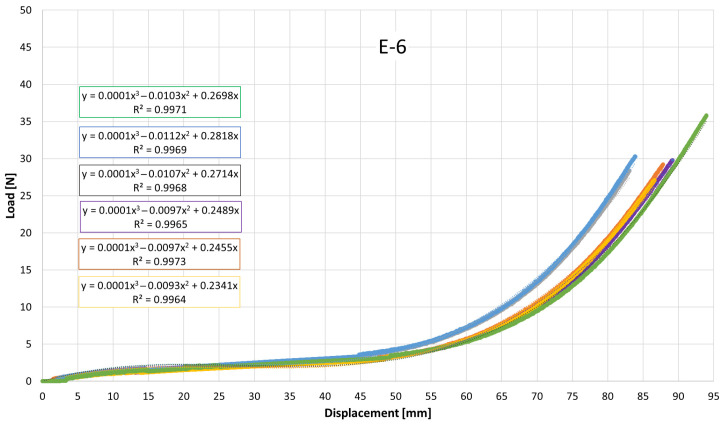
Load–displacement curve for test designation elastic, E-6.

**Figure 13:**
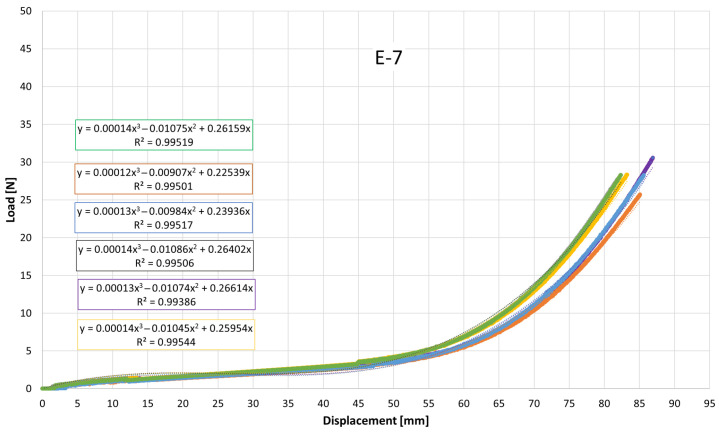
Load–displacement curve for test designation elastic, E-7.

**Figure 14:**
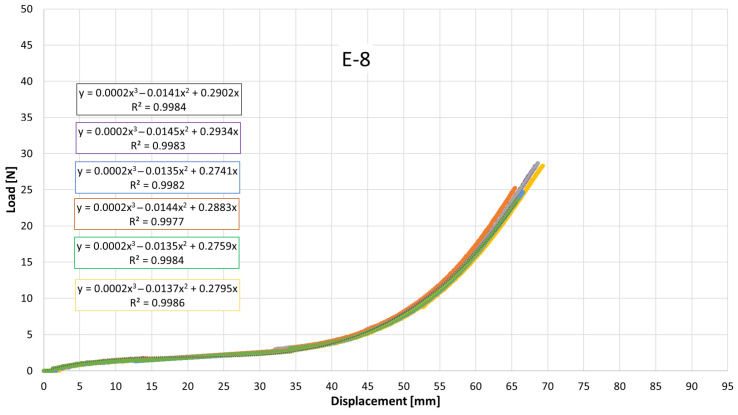
Load–displacement curve for test designation elastic, E-8.

**Figure 15:**
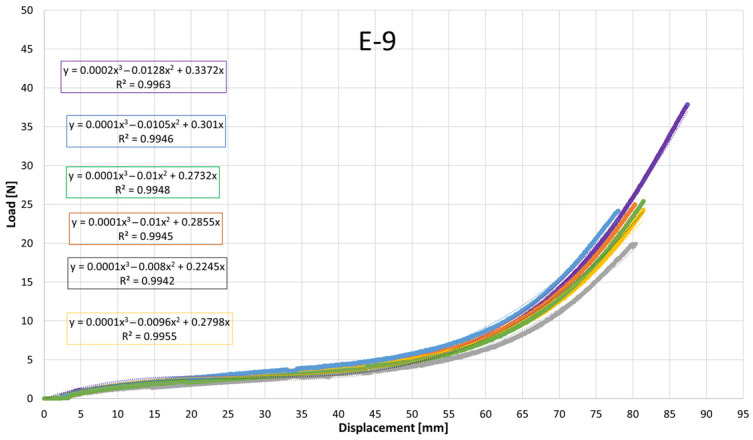
Load–displacement curve for test designation elastic, E-9.

Figure 7, Figure 8, Figure 9, Figure 10, Figure 11, Figure 12, Figure 13, Figure 14 and Figure 15 present the load–displacement responses of the nine intermaxillary elastic bands (E-1 to E-9) obtained from uniaxial tensile tests. For each band type, six replicate curves are shown, each corresponding to one of the six independent specimens tested, revealing the typical non-linear elastomeric response: an initial quasi-linear region up to approximately 8–10 mm of displacement, followed by a progressive stiffening and a steeper rise in load approaching the maximum extension [2].

The individual curves within each group overlap closely, indicating high repeatability of the tests and limited scatter, with slight divergence only near the peak-load region [3].

All elastics exhibit the characteristic non-linear tensile behaviour typical of elastomeric materials, with three distinct regions: (i) a nearly linear response at small displacements (<10 mm), (ii) a progressive curvature where the slope decreases slightly as the polymer chains align, and (iii) a pronounced stiffening at larger elongations approaching peak load.

The degree of stiffening and the slope of the initial region vary noticeably across the nine bands (E-1 to E-9), indicating differences in their initial stiffness and their resistance at higher extensions. For example, E-1, E-2, and E-3 display steeper initial slopes and reach high forces at moderate displacements, consistent with their higher nominal force ratings, whereas E-5, E-6, and E-7 exhibit softer initial responses, allowing larger displacements before significant force build-up. Within each group, the six replicates show tight clustering of the curves, confirming good repeatability and batch consistency.

Minor divergence is observed only near the final stages of extension—typically above 70–80% of their total stretch—where manufacturing tolerances and local stress concentrations influence the onset of non-linear stiffening and peak force. These graphical observations demonstrate that the primary distinctions among elastics lie in their early-stage stiffness and in the magnitude and rate of force growth at large elongations [37]. To quantify these differences and to enable predictive use of the curves in clinical settings, the mean responses of each elastic band were subsequently fitted with cubic regression equations, as detailed in the following subsection.

Table 4 reports the average regression coefficients (a, b, c) ± standard deviation obtained for each elastic band. The cubic term a, which governs the degree of stiffening at high elongations, is generally small and positive for most bands (typically 10^−6^ to 6 × 10^−4^, consistent with the gradual non-linear increase in force observed in the experimental curves. E-1 exhibited the highest a coefficient (0.0006), indicating a slightly stronger stiffening at large displacements, whereas E-4 and E-5 showed negligible cubic contribution (≈0.0000), suggesting a more linearized response over the tested range. The quadratic term b varies between approximately −0.03 and +0.001, reflecting differences in the curvature of the mid-displacement regime. Most bands present negative b values (e.g., E-1: −0.0203; E-2: −0.0307), which implies an initial reduction in slope before the cubic stiffening becomes dominant. Conversely, E-4 and E-5 show slightly positive b values (0.0013 and 0.0009), indicating a more gradual, nearly linear increase in stiffness. The linear term c, which is closely related to the initial stiffness of the band, spans from ≈0.024 N/mm (E-5) to ≈0.42 N/mm (E-1 and E-2). Bands with higher c values (E-1, E-2, E-3) therefore exhibit a steeper initial load rise with elongation, whereas bands such as E-5 show a considerably softer initial response. The standard deviations of the coefficients are generally low, particularly for c (≤0.05) and b (≤0.005 for most bands), indicating good repeatability of the regression fits across the six replicates for each band. The relatively larger scatter in a for some bands (e.g., E-2: 0.0024 ± 0.0034) is consistent with the increased experimental variability observed in the high-strain regime, where small differences in material properties become more pronounced. Overall, the cubic regression successfully captures the non-linear tensile behavior of all elastic bands and provides an analytical tool that enables clinicians and researchers to estimate the force delivered at any given intermaxillary displacement.

The mechanical characterization described above provides not only a scientific understanding of the tensile behavior of the elastic bands but also a practical resource for healthcare professionals, particularly orthodontists. Such information is essential for evidence-based clinical decision-making: relying solely on the manufacturer’s nominal classification (e.g., “light,” “medium,” and “heavy”) overlooks the product’s actual mechanical response and the inherent batch-to-batch variability.

**Figure 16:**
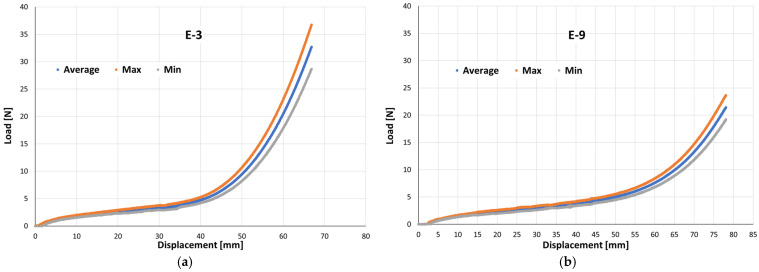
Load–displacement response of intermaxillary elastic bands obtained from six replicate tensile tests. The blue curve shows the mean force (N)—displacement (mm) behavior, while the orange and grey curves indicate the maximum and minimum experimental envelopes, respectively; (**a**) E-3 and (**b**) E-9.

The mean curve reflects the typical expected response, whereas the upper and lower envelopes quantify the experimental dispersion, thus defining the maximum and minimum forces likely to be encountered in practice. To illustrate the high repeatability of the experimental tests, Figure 16a,b show the load–displacement responses for elastic bands E-3 and E-9, including the mean response (blue curve) and the maximum and minimum envelopes (orange and grey curves, respectively) obtained from the six tested independent specimens. These plots demonstrate the limited scatter among specimens within the same elastic type, particularly across the clinically relevant displacement range.

**Figure 17:**
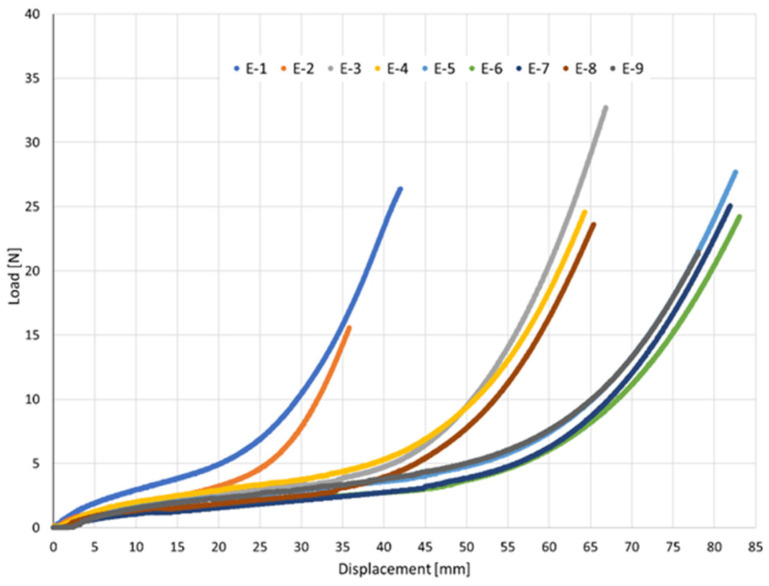
Mean load–displacement curves of the nine intermaxillary elastic bands (E-1 to E-9) obtained from each of the six replicate tensile tests.

This comparison provides clinicians with an at-a-glance view of how force builds with displacement for each band, thereby supporting the selection of the most suitable elastic to achieve the desired orthodontic loading. Figure 17 presents the mean load–displacement curves of all nine elastic bands (E-1 to E-9), enabling direct comparison of their mechanical responses.

The curves highlight substantial differences in stiffness and elongation capacity among the elastics: E-1 and E-2 show steeper force rises at short displacements (<40 mm); E-3, E-4, and E-8 display intermediate behavior; E-5, E-6, E-7, and E-9 allow longer displacements (>80 mm) with more gradual force build-up.

Despite the general similarity in the overall non-linear shape, the curves reveal clear distinctions in both the maximum displacement reached and the magnitude of the applied forces, which allow the elastics to be organized into functional groups [4,36]:

- **Group I—Short-displacement, high-stiffness elastics (E-1 and E-2):** These bands exhibit a steep force increase within relatively small displacements (<40 mm). E-1 reaches ≈ 27 N at ≈43 mm, while E-2 reaches ≈ 16 N at ≈36 mm. Their behaviour reflects higher stiffness, making them suitable when strong forces are required over short spans.
- **Group II—Intermediate-displacement, moderate-stiffness elastics (E-3, E-4, E-8):** These elastics extend further (≈65–70 mm) before reaching maximum load, with forces in the range of 26–36 N. Their curves show a more gradual stiffening, offering a balance between elongation capacity and force delivery.
- **Group III—Long-displacement, high-capacity elastics (E-5, E-6, E-7, E-9):** These bands sustain large elongations (>80 mm) with peak forces in the range of 26–36 N. E-5 and E-6 show the highest forces at maximum stretch, whereas E-7 and E-9 reach comparable elongations but with slightly lower peak forces. This group is most appropriate for applications requiring long intermaxillary movements with progressive force build-up.

This comparative overview demonstrates that, while all elastics share the same overall elastomeric response, their stiffness and extension capacities differ significantly. Organizing them into functional groups provides clinicians with a practical framework for selecting the optimal elastic type to deliver the desired orthodontic force–displacement behavior with greater predictability and patient safety.

### 3.3. Stress–Strain Representation

While the load–displacement curves presented in the previous sections provide insight into the overall mechanical behavior of the elastic bands, these responses are influenced by the initial geometry of each product, particularly the cross-sectional area and the initial gauge length.

To enable a direct comparison of the intrinsic properties of the elastomeric materials, the experimental force–displacement data were normalized to obtain engineering stress and strain. This normalization transforms the measured forces into stress (MPa) by dividing by the initial cross-sectional area, and the measured displacements into strain (%) by relating the elongation to the initial length of the specimen.

The resulting stress–strain curves allow a fair assessment of the material’s stiffness (initial modulus) and strain-hardening behavior at large deformations, irrespective of the original band size. Such a representation is particularly valuable for clinical applications: it highlights whether the differences observed among the bands are due to the material formulation or simply to geometrical factors.

**Figure 18:**
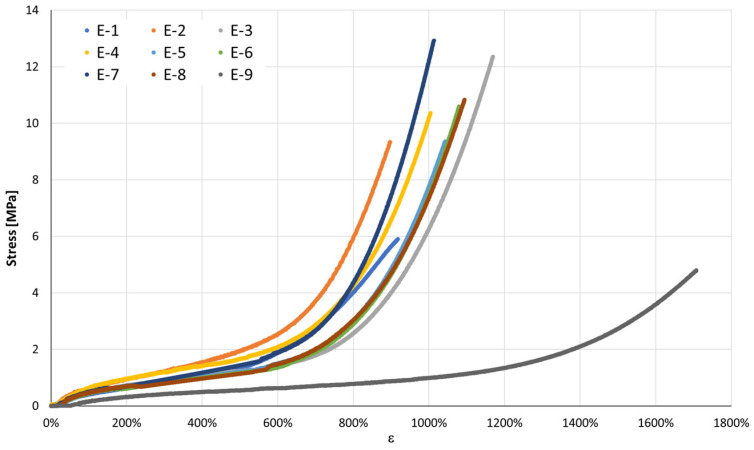
Mean stress–strain curves of the nine intermaxillary elastic bands (E-1 to E-9) derived from averaged tensile tests after normalization by cross-sectional area and initial gauge length.

Figure 18 displays the mean stress–strain curves of the nine intermaxillary elastic bands (E-1 to E-9), derived from the averaged force–displacement data and normalized by the initial cross-sectional area and gauge length of each band. This, normalization removes geometry effects and reveals the intrinsic material response of the elastomers, enabling direct comparison of their stiffness, strain-hardening behavior [16], peak stress, and strain capacity, which are key parameters in orthodontic force management.

#### 3.3.1. General Behavior

All elastics exhibit the typical non-linear response of cross-linked elastomers, beginning with a low-strain region (up to≈ 200–300% strain) with an approximately linear increase in stress, followed by a transition zone in which the slope gradually steepens as the polymer chains align, and culminating in a pronounced strain-induced hardening beyond ≈600–800% strain, leading up to peak stress.

#### 3.3.2. Initial Modulus (Small-Strain Stiffness)

Differences in the initial slope reveal that some elastomers are inherently stiffer at small deformations: E-2, E-4, and E-8 exhibit the steepest initial rise, indicating higher small-strain modulus; E-3 and E-9 show the lowest initial stiffness, with a much flatter initial region before significant stress build-up; the remaining bands (E-1, E-5, E-6, E-7) present intermediate initial slopes.

These findings highlight that the high apparent stiffness of some elastics in the raw force–displacement plots (e.g., E-1) was partly due to smaller cross-sections rather than intrinsically higher modulus [3].

#### 3.3.3. Large-Strain Hardening and Peak Stress

At large deformations, all bands demonstrate pronounced hardening: the highest peak stresses are reached by E-6 (≈13.8 MPa) and E-7 (≈14.4 MPa), reflecting strong network resistance at extreme stretches; E-3, E-4, E-5, and E-8 form an intermediate group with peak stresses ≈ 11–12.5 MPa, maintaining good extensibility with robust strength; E-1, E-2 and E-9 exhibit lower peak stresses (≈4–6 MPa), offering gentler force response even at high elongations.

#### 3.3.4. Strain Capacity

All elastomers sustain high extensibility, with peak strains exceeding 900% for most bands and reaching ≈ 1750% for E-9. Notably, E-9 combines very high extensibility with low stress, which may be advantageous for long-range, low-force clinical applications [38].

## 4. Discussion

Intermaxillary elastics are widely used in orthodontics as auxiliary devices to modify occlusal relationships and control mandibular biomechanics [5]. Their action relies on continuous forces that induce compressive and tensile stresses in the periodontal ligament, stimulating osteoblastic and osteoclastic activity [38]. However, improper or prolonged use can compromise periodontal health, potentially causing inflammation, abscesses, bone loss, and alterations in keratinized gingiva or the mucogingival junction [7]. It is worth noting that intermaxillary elastics do not maintain a constant force over time, undergoing progressive mechanical degradation during clinical use [11,12,13]. In the present study, all evaluated elastics exhibited non-linear elastomeric behaviour with an initial quasi-linear region followed by gradual stiffening as polymer chains align, culminating in pronounced strain-hardening at large deformations, consistent with previous reports [11,12,13,39].

Consistent with previous research, the elastics evaluated in this study demonstrated the characteristic nonlinear elastomeric behavior. While most prior investigations have primarily focused on force decay over time under constant extension, reporting progressive reduction in force due to stress relaxation and environmental exposure [2] the present study was designed to assess the intrinsic mechanical properties through monotonic tensile testing until rupture. This approach enabled the characterization of stiffness, maximum force, and energy-related parameters that reflect the material’s structural integrity and load-bearing capacity. However, it is important to emphasize that time-dependent behavior, including force degradation and stress relaxation under sustained clinical conditions, was not evaluated in the present protocol. Therefore, the findings should be interpreted as representative of the immediate mechanical response rather than the long-term force delivery profile.

It should be noted that elastomeric materials exhibit strain-rate-dependent viscoelastic behavior. Mechanical parameters such as peak stress, stiffness, and energy absorption may vary with loading rate. The present study was conducted under quasi-static monotonic conditions (3.3 mm/min), which may differ from the dynamic and intermittent loading experienced intraorally. Therefore, the reported values should be interpreted as intrinsic material properties under controlled laboratory conditions rather than direct representations of clinical force delivery under functional strain rates.

These differences are particularly relevant for clinical application, as they imply distinct force-delivery profiles under similar activation distances, directly influencing treatment efficiency and patient comfort. While load–displacement curves provide valuable information on the overall mechanical response, they remain inherently dependent on elastic geometry. In this context, the stress–strain representation enables a normalized, geometry-independent comparison of intrinsic material behavior, allowing a clearer distinction between size effects and material formulation. Consequently, the inclusion of energy-based parameters, such as strain energy density at rupture, provides an additional descriptor of mechanical performance, reflecting the material’s capacity to absorb and sustain energy during large deformations.

The results demonstrated that geometry, particularly elastic size, is a decisive factor in mechanical response. Elastics E-5, E-6, and E-7, despite originating from different manufacturers, exhibited comparable mechanical behavior and high energy absorption, reflecting equivalent size and strength. Similarly, E-1 and E-2 showed similar profiles despite different nominal strength ratings, suggesting that manufacturer-designated size is a more reliable predictor of mechanical behavior than nominal force classification. Elastics E-3, E-4, and E-8, although originating from different brands and assigned distinct strength ratings, demonstrated comparable displacement behavior, further indicating that these elastics are effectively equivalent in size. These findings reinforce the crucial role of geometry parameters, particularly elastic size, in dictating the mechanical response of intermaxillary elastics [2,40]. Complementing these findings, Dubovská et al. reported in vitro testing of 3/16” medium elastics from five manufacturers, revealing notable differences in initial force and force degradation over time, with the greatest reduction occurring within the first two hours and variability in stability among brands, highlighting the importance of clinicians’ understanding the specific mechanical properties of the elastics they prescribe [39].

Stress–strain analysis provided additional insight beyond conventional load–displacement curves, revealing substantial differences in normalized material properties between bands. For clinical applications requiring firmer forces over short activation distances, elastics E-2, E-4, and E-8 show superior performance due to their high initial modulus. On the other hand, when intense forces accompanied by large elongations are required, larger elastics, such as E-6 and E-7, are the most appropriate choice. For situations where gentle forces applied over long displacements are desired, the E-9 elastic is preferable due to its high deformability and reduced maximum tension. These scenario-specific interpretations are particularly relevant in implant-related biomechanics and pre-prosthetic orthodontics, such as controlled extrusion for ridge augmentation or vertical site development, where predictable force delivery and tissue protection are critical. Therefore, normalized stress–strain curves thus offer a size-independent framework for evidence-based elastic selection, enabling predictable force delivery at specific elongation ranges [3].

Consistent with prior studies, manufacturer labels such as ‘light,’ ‘medium,’ and ‘strong’ did not reliably correspond to actual mechanical behavior [10,39]. It is emphasized that manufacturer force ratings typically refer to a single stretch condition that was not disclosed and therefore do not fully represent the complete mechanical behavior measured here. The experimental classification obtained in this study (based on stiffness, displacement, and force-deformation response) often did not match the force indicated by the manufacturer. Elastics classified as medium or strong sometimes behaved similarly to weaker elastics, while some “light” elastics generated higher forces than expected. These discrepancies arise because the force in ounces provided by the manufacturer, usually refers to only a single point of stretch, which does not represent the entire mechanical curve or real clinical situations [10]. These inconsistencies have an important clinical impact: relying solely on the commercial label can lead to the use of inappropriate forces, either through underloading (slow movement) or overloading (pain, tissue damage). Mapping the measured force–displacement behavior to clinical scenarios allows clinicians to select elastics that deliver predictable forces for specific applications, enhancing patient safety and treatment orthodontic efficiency. The mean and envelope curves presented here allow clinicians to estimate the full range of forces at clinically relevant displacements, improving predictability and patient safety. Statistical analyses identified three functional groups based on mechanical behavior, clarifying which elastics offer greater energy absorption, extensibility, or stress capacity, thereby supporting data-driven selection aligned with clinical force requirements.

Despite providing valuable insights into the mechanical behavior of intermaxillary elastics, the present study has several limitations. Testing was conducted in vitro under controlled, dry conditions with constant force application, which does not fully replicate the dynamic mechanical fluctuations experienced in the oral cavity due to chewing, speaking, or mandibular movements. Cross-sectional dimensions were treated as constant within each product, and only a limited number of replicates were assessed, which may underestimate batch-to-batch variability. Furthermore, the absence of cyclic loading, fatigue assessment, intraoral simulation, exposure to saliva, temperature cycling, and viscoelastic relaxation over time fundamentally limits the direct translation of these findings to clinical orthodontic use. Moreover, future studies should systematically evaluate elastics with identical nominal dimensions but from different brands and materials, to distinguish the contributions of material composition from geometric factors.

It is recognized that elastomeric materials may exhibit Mullins softening and viscoelastic effects during initial loading cycles. Since no mechanical preconditioning was performed, the initial loading segment of the force-displacement curves may reflect first-cycle material response. Future studies incorporating cyclic preconditioning and repeated loading protocols would provide additional insight into the functional intraoral behavior of orthodontic elastics.

The present findings should be interpreted as a standardized static mechanical comparison under controlled conditions, describing intrinsic force–elongation behavior rather than predicting long-term intraoral degradation, fatigue, or biological response.

Previous in vitro work has shown that intraoral thermal fluctuations can alter the mechanical responses of orthodontic materials, reinforcing the need for future elastic testing to include thermocycling and saliva exposure to better approximate clinical conditions [41]. Consequently, future studies should incorporate larger sample sizes, in vitro dynamic protocols, thermocycling, artificial saliva, pH variations, long-term aging, latex versus non-latex comparative fatigue testing, multi-vector loading, clinically realistic anchorage systems, and correlations with in vivo force decay and biological response markers to more accurately predict clinical performance. Clinically, the results of this study must be interpreted within the constraints of the study’s in vitro nature, the use of a single product lot, and the absence of time-dependent relaxation testing. Consequently, these findings should not be viewed as a guarantee of direct clinical equivalence, but rather as an evidence-based guide. Instead of relying solely on manufacturer-designated force ratings, orthodontists are encouraged to consider the mechanical parameters identified—such as initial stiffness, peak stress, and normalized stress–strain behavior—to better estimate the force–elongation profile for each patient. Adopting this qualified, data-driven approach can help refine the predictability of orthodontic loading and optimize tooth movement while maintaining a focus on patient safety.

Despite providing a comprehensive mechanical characterization under standardized conditions, several experimental limitations must be acknowledged.

First, testing was performed under monotonic quasi-static uniaxial tensile loading at a constant crosshead speed (3.3 mm/min), following ISO 37:2011. This configuration does not replicate the complex intraoral environment, where elastics are subjected to cyclic loading, intermittent activation, multi-vector force components, and mandibular dynamics. The absence of cyclic fatigue testing, stress-relaxation protocols, creep analysis, or dynamic loading represents a limitation when extrapolating results to long-term clinical force delivery.

Second, environmental simulation was limited to controlled laboratory conditions (22 °C, 54% RH). No artificial saliva immersion, thermocycling, pH variation, or thermo-mechanical aging was performed. Because intraoral force degradation is influenced by moisture, enzymatic activity, temperature fluctuations, and dietary factors, the present findings reflect intrinsic mechanical behavior rather than in vivo degradation kinetics.

Third, the determination of initial gauge length (L_0_) was based on a standardized preload of 0.05 N. Although this procedure improves repeatability by eliminating slack, it remains dependent on instrument resolution and load-cell sensitivity. No independent reproducibility validation of L_0_ measurement was conducted.

Fourth, only six specimens per group were tested, and no a priori power analysis was performed. While effect sizes were large and statistical significance was achieved, the limited sample size restricts generalizability and may underestimate inter-batch variability.

Fifth, all specimens were obtained from a single production lot per elastic type. Batch-to-batch variability was not assessed, limiting conclusions about manufacturing consistency.

Sixth, geometry and material composition were not experimentally decoupled. Elastics of different sizes inherently differ in their cross-sectional dimensions, potentially confounding geometric scaling with intrinsic material formulation. Although stress–strain normalization mitigates size effects, complete isolation of material composition would require testing identical geometries fabricated from different materials and vice versa.

Finally, cross-sectional area was treated as constant within each product type based on pre-test optical microscopy measurements. Although dimensional variability was small, this assumption may introduce systematic uncertainty in stress calculations.

Although effect sizes were large, no a priori power analysis was conducted, and additional specimens and multi-batch sampling would improve statistical power and generalizability.

Future investigations should incorporate cyclic fatigue protocols, stress-relaxation testing, artificial saliva immersion, thermocycling, multi-batch sampling, increased specimen numbers supported by power analysis, and controlled geometry–material separation designs. Such improvements would enhance translational relevance and strengthen predictive modeling of intraoral elastic performance.

## 5. Conclusions

The present study shows that intermaxillary elastics’ mechanical behavior is strongly influenced by size and cannot be reliably predicted from manufacturer labels alone. All elastics exhibited non-linear behavior, with initial stiffness followed by strain-hardening, while stress–strain analysis revealed substantial differences in normalized properties. Classification into functional groups based on stiffness, peak stress, and extensibility provides a size-independent framework for evidence-based selection. Although limited to laboratory conditions, these results provide valuable insights into elastic performance, highlighting discrepancies with manufacturer force ratings and supporting more informed, data-driven decisions in clinical practice.

## Figures and Tables

**Table 1:** Specifications of intermaxillary elastics used in the study.

Test Designation	Material	Expiry Date	Manufacturer Classification	Force			Elastic Size	
				ozf	Newton [N]	gf	Size [Inch]	Diameter [mm]
E-1	Latex	17 January 2026	Strong pull	6.5	1.8	184	1/8	3.2
E-2	Latex	13 April 2027	-------	4.5	1.25	130	1/8	3.2
E-3	Latex	8 September 2026	-------	6	1.67	170	3/16	4.8
E-4	Latex	17 April 2026	Heavy	6.5	1.8	184	3/16	4.8
E-5	Latex	16 February 2026	Heavy	6.5	1.8	184	1/4	6.4
E-6	Latex	12 February 2025	Medium	4.5	1.25	130	1/4	6.4
E-7	Latex	29 July 2026	-------	3.5	0.97	100	1/4	6.4
E-8	Latex	16 March 2026	Medium	4.5	1.25	130	3/16	4.8
E-9	Latex	28 February 2027	Strong pull	6.5	1.8	184	1/4	6.4

**Table 2:** Physical measurements of elastics obtained by the optical microscope.

Test Designation	Elastic Thickness (t) [mm]	Elastic Height (h) [mm]	Section (A_0_) [mm^2^]	ϕ int [mm]	ϕ ext [mm]
E-1	1.4	1.6	2.2	2.9	5.8
E-2	0.8	1.0	0.8	3.0	4.7
E-3	0.8	1.6	1.3	4.6	6.3
E-4	1.3	1.0	1.3	4.8	7.4
E-5	1.3	1.2	1.5	6.3	8.9
E-6	0.9	1.3	1.1	6.4	8.1
E-7	1.1	0.9	1.0	6.6	8.6
E-8	1.0	1.1	1.1	4.7	6.7
E-9	1.3	1.3	1.7	6.2	8.8

ϕ int—internal diameter; ϕ ext—external diameter.

**Table 3:** Average mechanical properties (mean ± standard deviation, *n* = 6) of the nine intermaxillary elastic bands (E-1 to E-9) obtained under uniaxial tensile loading. Parameters include energy absorbed up to rupture (work to rupture W_R_), displacement at peak load, peak load, strain at peak load (ε), and corresponding peak stress.

Designation	Specific Rupture Work [J/m^2^] ± STD	Displacement at Peak Load [mm] ± STD	Peak Load [N] ± STD	ε [%] ± STD	Peak Stress [MPa] ± STD
E-1	502.1 ± 11.6	43.2 ± 0.9	27.3 ± 2.4	945.3 ± 19.9	6.2 ± 0.5
E-2	502.1 ± 11.6	36.5 ± 0.8	16.0 ± 2.0	917.2 ± 18.9	4.4 ± 1.3
E-3	1194.5 ± 72.2	67.8 ± 1.0	33.5 ± 2.7	1187.4 ± 17.8	12.6 ± 1.0
E-4	1311.3 ± 94.6	68.6 ± 2.3	30.5 ± 5.2	1070.8 ± 36.2	12.2 ± 1.5
E-5	1152.0 ± 90.9	88.2 ± 1.6	35.9 ± 3.08	1114.5 ± 20.3	12.4 ± 1.1
E-6	1253.3 ± 94.7	88.4 ± 4.2	30.2 ± 2.8	1149.5 ± 54.7	13.8 ± 1.4
E-7	1281.6 ± 37.9	80.4 ± 1.4	27.8 ± 1.0	1040.1 ± 17.6	14.4 ± 0.5
E-8	1064.5 ± 51.5	66.6 ± 1.3	25.9 ± 1.9	1115.1 ± 21.4	11.5 ± 0.7
E-9	732.6 ± 79.1	80.1 ± 1.5	23.83 ± 1.8	1752.8 ± 33.2	5.54 ± 0.1

Note: Full post-hoc pairwise comparison tables (Tukey HSD, adjusted *p*-values and confidence intervals) are provided as Supplementary Materials.

**Table 4:** Average cubic regression coefficients (a, b, c) ± standard deviation (STD) for the mean load–displacement curves of the nine intermaxillary elastic bands (E-1 to E-9). The fitted function from Equation (5), where F is the load (N) and x is the displacement (mm).

Designation	Regression Coefficients		
	a	b	c
E-1	0.0006	−0.0203	0.4211
STD	0.000111	0.00494	0.0521
E-2	0.0024	−0.0307	0.4142
STD	0.00338	0.00394	0.048
E-3	0.0003	−0.0197	0.4016
STD	0.0000373	0.00212	0.042
E-4	0	0.0013	0.0298
STD	0.0000197	0.00126	0.0298
E-5	0	0.0009	0.0243
STD	0.0000471	0.000867	0.0243
E-6	0.0001	−0.0102	0.2586
STD	0	0.000653	0.0168
E-7	0.0001	−0.0103	0.2527
STD	0.00000745	0.000628	0.015
E-8	0.0002	−0.014	0.2836
STD	0	0.000407	0.00739
E-9	0.0001	−0.0102	0.2835
STD	0.0000373	0.00142	0.0337

## Data Availability

The original contributions presented in the study are included in the article/Supplementary Materials, further inquiries can be directed to the corresponding author.

## Supplementary Materials

The following supporting information can be downloaded at: https://www.mdpi.com/article/10.3390/jfb17030117/s1.

## Abbreviations

The following abbreviations are used in this manuscript:

ANOVA	One-way analysis of variance
ISO	International Organization for Standardization
STD	Standard deviation
W_R_	Work to Rupture
